# Development of an AVF Stenosis Assessment Tool for Hemodialysis Patients Using Robotic Ultrasound System

**DOI:** 10.3390/mi9020051

**Published:** 2018-01-29

**Authors:** Yi-Chun Du, Jheng-Bang Shih, Ming-Jui Wu, Chung-Yi Chiou

**Affiliations:** 1 Department of Electrical Engineering, Southern Taiwan University of Science and Technology, No. 1, Nan-Tai Street, Yungkang Dist., Tainan 71005, Taiwan; ma320211@stust.edu.tw (J.-B.S.); wmr216@yahoo.com.tw (M.-J.W.); snowymoon1120@gmail.com (C.-Y.C.); 2 Department of Internal Medicine, Kaohsiung Veterans General Hospital Tainan Branch, Tainan 71051, Taiwan

**Keywords:** Hemodialysis (HD), arteriovenous fistula (AVF), robotic ultrasound system (RUS), degree of stenosis (DOS)

## Abstract

With the aging population and lifestyle changes, the number of hemodialysis (HD) patients increases year by year. The arteriovenous fistula (AVF) is the gold standard vascular access used to access the blood for HD treatment. Since the status of stenosis affects HD efficiency, current clinical practices usually use a Doppler ultrasound imaging system to assess the parameters of the stenosis, such as the degree of stenosis (DOS). Unfortunately, this is a very time-consuming task. Furthermore, it is difficult to stably maintain the ultrasound probe for a prolonged period to give doctors clearer or reproducible images. In this study, a robotic ultrasound system (RUS) with ultrasound sequential imaging analysis was designed to evaluate the DOS of the AVF. The sequential imaging analysis was capable of image smoothing and vessel boundary detection. It enabled clinicians to mark the thickness of the plaque for further processing. Finally, the system was used to reconstruct 3D models of fistulas and calculated the DOS for clinical assessment. We also designed a pressure sensing module attached to the ultrasound probe to prevent the probe from coming loose, vibrating, and exerting abnormal pressure on the skin. In the phantom test, the results showed that the error of the DOS that was calculated by RUS was less than 3%. The results of clinical trials obtained from ten patients show that the error between the RUS and clinicians’ measurement was about 10% and had a highly linear correlation (*R* Square > 0.95). In addition, the reproducibility error was about 3% and could effectively save about 46% of the time during clinical examinations.

## 1. Introduction

Hemodialysis (HD) is a therapeutic method that helps excrete neurotoxins and water from patients with severe kidney problem to stabilize their life. Over two million people worldwide currently receive dialysis treatment or undergo kidney transplant to stay alive. It is expected that this number will have an annual growth rate of 8% [1]. Dialysis patients in Taiwan have already exceeded 80 thousand people; the largest number in terms of population ratio in the world [2]. The process of HD requires treatment through fistulas. Fistulas are divided into two kinds, namely arteriovenous fistula (AVF) and arteriovenous graft (AVG). AVF has a longer lifespan and is currently the most widely used permanent vascular access, requiring surgery to connect the artery to the vein. When the vein receives the impact from the arterial blood flow, the vein wall thickens and a fistula is formed. Thus, the vein would be able to withstand repeated needle puncture and not be easily damaged during HD. In clinical experience, during the first year of dialysis, a blockage rate of about twenty percent was observed in patients using AVF, while a blockage rate of 30% to 40% was detected in AVG patients. Fistula occlusion could cause severe and fatal complications such as congestive heart failure, ischemia neuropathy, infection, etc., [3,4,5].

Therefore, the fistula is a very important artificial blood vessel for dialysis patients and is sometimes referred to as the lifeline. Current clinical practices usually use ultrasound images to assess fistula occlusion [6,7]. The ultrasound system is non-invasive, radiation-free, and economical; therefore, it is a popular imaging equipment tool in medical institutions. Although ultrasound imaging in clinical usage is extremely high, nevertheless, it relies on the operational experience of doctors. Forceful exertions and repeated use of the ultrasound probe at a certain angle are important procedures during ultrasound examination [8]. Moreover, certain positions of the probe need to be maintained stably for a prolonged period in order to get a clear image for examination. Long-term ultrasound operation is a common cause of occupational injury, such as shoulder pain among doctors [9]. Robotic manipulators have the advantages of being accurate, consistent, and stable. They also reduce instances of contamination. They can be employed to improve the acquisition and use of real-time medical imaging systems and make them more convenient [10,11].

The American company Intuitive Surgical, Inc. (Sunnyvale, CA, USA) developed a special robot, the da Vinci^®^ Surgical System, for minimally invasive surgeries. The da Vinci robot has more than four robotic arms with six degrees of freedom (6-DOF). In addition, the force feedback design allows physicians to precisely conduct minimally invasive surgeries. For example, with the help of the da Vinci surgical system, a precision of less than 0.1 mm can be achieved on vascular suturing [11]. In 2013, Sutherland and Garnette, R. et al. developed the NeuroArm, a robotic neurosurgical device that uses magnetic resonance imaging (MRI) for an instantaneous surgical guide. This system also provides two 3D cameras for obtaining real-time 3D images of patients. Injection, suturing, burning, cutting, and other surgical procedures are performed in coordination with MRI images [12]. Many research groups had specialized application on the flexibility of robot-assisted diagnostic ultrasound. The results indicated that the robotic arms were able to lithely and skillfully execute the given instructions. Furthermore, they had a high potential to be extended for use in many surgical manipulations. These studies showed that the medical robotic arm will become an integral part of clinical medicine in the near future [13]. The robotic ultrasound system (RUS) can be defined as the combination of ultrasound imaging with a robotic system in medical application [14]. Since 1999, scholars have used RUS for automatic carotid artery detection. The robotic arm used in the ultrasound system was able to move freely and easily. In 2011, Nakadate, R. et al. proposed an automatic RUS could track the carotid artery on the skin for the diagnostic system. The pressure feedback from the ultrasound probe or computer vision from the camera could be used to maintain the image quality and ensure patient safety of the RUS. Moreover, the robotic arm was able to slightly control the movement of the ultrasound probe on the target plane to get better images [15]. In 2013, Mustafa, B. et al. developed a remote RUS used in automatic scanning of the liver. The proposed RUS was combined with a camera to obtain surface skin images to automatically locate the position of the navel and the nipples. The information was used to analyze the best position for scanning the liver. This system was able to obtain an accuracy of up to 94% [16]. In 2014, Janvier, M. A. et al. used image processing to reconstruct a traditional 2D B mode ultrasound image into a 3D artery image for clinical assessment of lower limb arterial stenosis, which led to the a successful treatment program [17]. In 2012, Chadli, S. et al. combined RUS and telemedicine techniques to assist routine ultrasound examination in remote districts. This system also included a pressure sensing function and five-axis accelerometer for real-time detection and feedback of probe status to ensure image quality and patient safety [18].

The foregoing studies show that the RUS has a high stability and high consistency in ultrasound examination [13,14,15,16,17,18]. When coupled with automatic image processing, it can be used to analyze 3D structures. In this study, we proposed a RUS for current clinical practices in assessing fistula occlusions. The system can be applied to the automatic routine examination of fistulas in HD patients. Unlike carotid arteries, the fistula pathways are more intricate; therefore, we designed three scan methods to identify the ultrasound scan path in different fistula pathways. In order to reduce the system cost and the difficulty of system integration, the system was designed base on a 3-axis robotic arm and is capable of capturing ultrasound images from traditional video graphics array (VGA) and high definition multimedia interface (HDMI) video ports, the currently popular display signal ports in a medical ultrasound system. After clinicians set the scanning pathway, the RUS can scan and obtain good quality ultrasound images of the fistula. It can also record the distance and blockage position.

## 2. Methodology

### 2.1. Architecture of the Robotic Ultrasound System (RUS)

**Figure 1:**
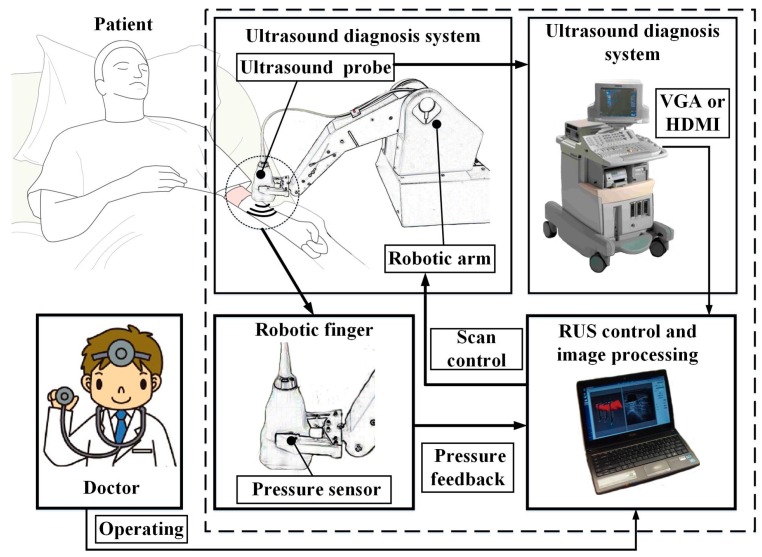
Schematic diagram of the robotic ultrasound system (RUS).

The RUS architecture consisted of three parts: the robotic arm system, the ultrasound imaging system, and the image capture system, as shown in Figure 1. The robotic arm system provided a five-axis movement for scanning. Clinicians mainly examined if the occlusion site was an Arterial Anastomosis Site (A site), a Venous Anastomosis Site (V site), or a Loop Site (L site). Since the A site is the artery collision point, it has the largest diameter in the fistula. Positions that are often blocked are on the V site and L site. Therefore, this system set these three sites as the scan target. In addition, during automatic scanning, to help stabilize the movement of the ultrasound probe, a pressure sensing system was installed on the robotic finger to prevent the ultrasound probe from becoming loose while scanning. This allowed the system to get the best image quality of the fistula. Existing clinical models of ultrasound systems used in hospitals were used for this system. In order to be compatible with different models when storing images for analysis, this system used VGA and HDMI video capture cards to obtain ultrasound images every second. During system analysis of fistula condition, each image captured during robotic arm motion and scanning was transmitted to the image capture control system for analysis.

**Figure 2:**
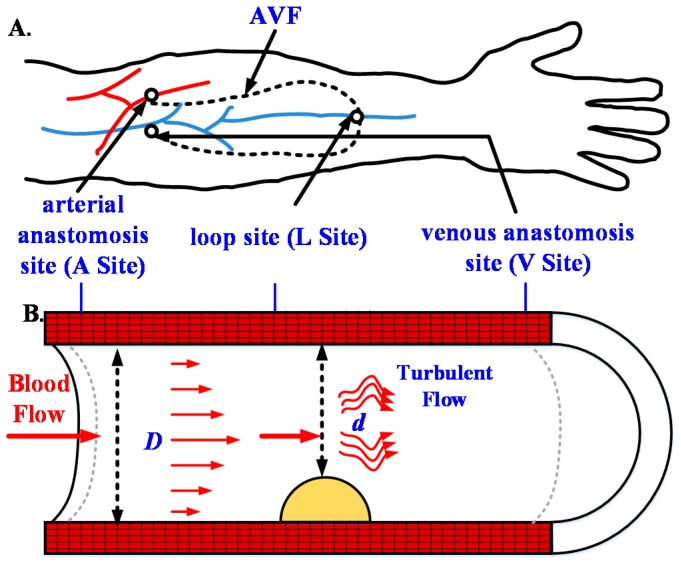
Schematic diagram of (**A**) the arteriovenous fistula (AVF) and (**B**) the degree of stenosis (DOS) parameters.

As for the image capture control system, two references were used for clinical assessment of fistula occlusion, one was the normal diameter of the fistula *D* and the other was the thickness of plaque *d*. In current medical practices, these two parameters are calculated by the doctors after the manual capture of scan images. However, the present study used the RUS to automatically calculate these references. During semi-automatic scanning by the robotic arm, the captured image after each motion was analyzed. The fistula type was automatically encircled to calculate the degree of stenosis (DOS) according to Equation (1) to help doctors understand the patient’s fistula occlusion situation. The schematic diagram of the DOS in AVF was shown in Figure 2 [19].

(1) $$DOS\%=(1-\frac{d^{2}}{D^{2}})\times 100\%$$

### 2.2. Robotic Arm 

**Figure 3:**
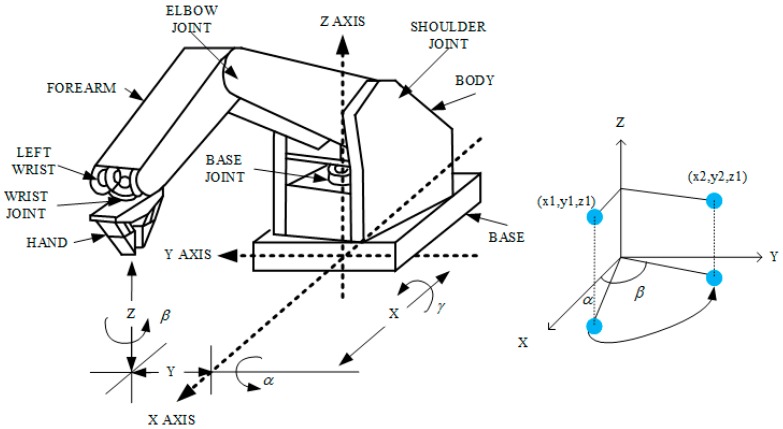
The five-axis robotic arm and degree of freedom of the control coordinate system.

The five-axis robotic arm used in this study was the Microbot Teachmover II (Questech, Inc., Farmington Hills, MI, USA). The robotic arm system was used with the RS232 serial (EIA, District of Columbia, WA, USA) communication with packet transmission rate set at 9600. This five-axis robotic arm was controlled by a motor and could move in the direction of the *x*, *y*, and *z*-axis, as shown in Figure 3. Furthermore, it had six degrees of freedom for motion, namely Base Motion: ±90°, Shoulder Motion: ±144°, −35°, Elbow Motion: ±0°, −149°, Wrist Roll: ±180°, Wrist Patch: ±90°, and Gripper Opening: 0–75 m [20].

For the robotic arm range of motion, the *x*-axis telescopic range was 30.48 cm, the *y*-axis rotation radius was 30.48 cm, and the *z*-axis radius was 44.45 cm. The setting was made with the robotic arm as the point of origin. The robotic arm scanning region had a length of 15 to 25 cm, a width of 40 to 50 cm, and a height of 10 to 15 cm. These regions of motion could fully cover the entire arm during an ultrasound scan. Moreover, the robotic arm did not require a large area for its movement; it just needed to scan the fistula in the arm. As for the system configuration, the motion of the robotic arm in each axis had a resolution of 0.025 cm. Its motion speed range was 0 to 17.8 cm/s. To obtain good ultrasound images, speed was set to 0.5 cm/s.

### 2.3. Pressure Sensing Module

The robotic finger had mechanical grip strength of 13 N, which was capable of steadily holding the ultrasound probe. However, in order to prevent the ultrasound probe from becoming loose during scans, exerting abnormal pressure on the skin and causing probe vibrations, a pressure sensing module was attached to the ultrasound probe connected to the robotic arm. The sensor, Uneo GD05-10N (Uneo, Inc., New Taipei, Taiwan), was made of flexible microelectromechanical systems (MEMS) that could measure the magnitude of the pressure through changes in resistance [21]. Its sensing range, size, and area were 0 to 10 N, 8.6 mm, and 5 mm, respectively. The module circuit was designed using Wheatstone’s electric bridge with an amplifier gain of 100. We also conducted some standard tests to find the regressive equation to transfer the voltage to the probe pressure on the skin. The microprocessor used was the CC2540 (Texas Instruments, Dallas, TX, USA) with 8 KB random access memory (RAM), capable of real-time recording of the pressure within 8 min at a 10 Hz sampling rate. The data could be transmitted to the RUS wirelessly through Bluetooth 4.0. The pressure information acted as a control signal for the regulation of the robotic grip. It revealed whether the ultrasound probe had become loose, produced vibrations, or showed another abnormal status.

### 2.4. Ultrasound Device

In order to reduce the difficulty of system integration, the proposed RUS could capture ultrasound images from traditional VGA and HDMI video ports of conventional ultrasound instruments. The ultrasound equipment utilized in this study was the HD11 (Philips, Eindhoven, The Netherlands). It is a traditional shared service-imaging workhorse. Being a very mature platform, it is stable, cost-effective, highly serviceable, and has good image quality. The transducer used was a broadband linear array with a frequency of 7.5 MHz. The linear ultrasound probe width was 38 mm, and scan depth could reach 8 cm, which was frequently used in clinical trials on the fistula examination.

### 2.5. Scanning Methods

**Figure 4:**
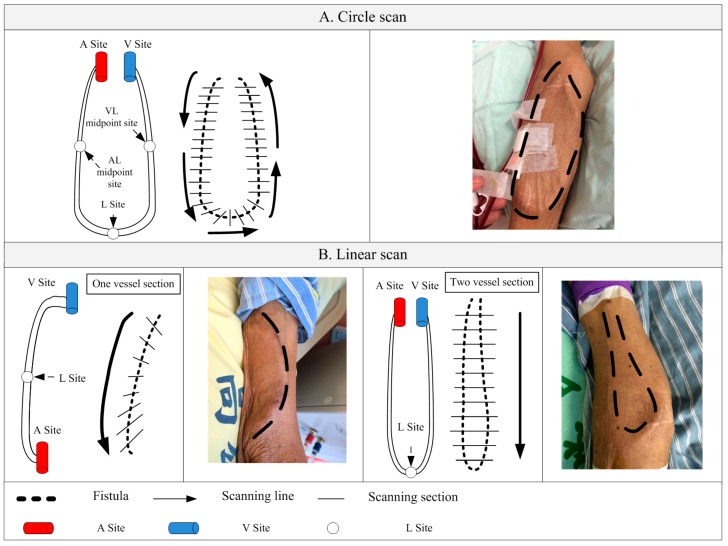
Two scanning modes (**A**) circle and (**B**) linear scan.

There are two most commonly seen types of fistulas in clinical dialysis, as shown in Figure 4. After discussions with clinicians, two methods of scanning were determined in this study, namely, circle scan (Figure 4A) and linear scan (Figure 4B). Scanning spots had to be provided for these two scanning methods in order to compute the scan path of the mechanical arm. The necessary scan spots set for the different scanning methods were the same, that was, the arterial anastomosis site (A site), venous anastomosis site (V site) and loop site (L site) of the AVF. Naturally, more spots could provide better assistance for the scan pathway setting in the robotic arm. However, in order to reduce the setting time in the proposed RUS, the user was required to input at least three points for the calculation of the pathway.

### 2.6. Scan Path Computation

**Figure 5:**
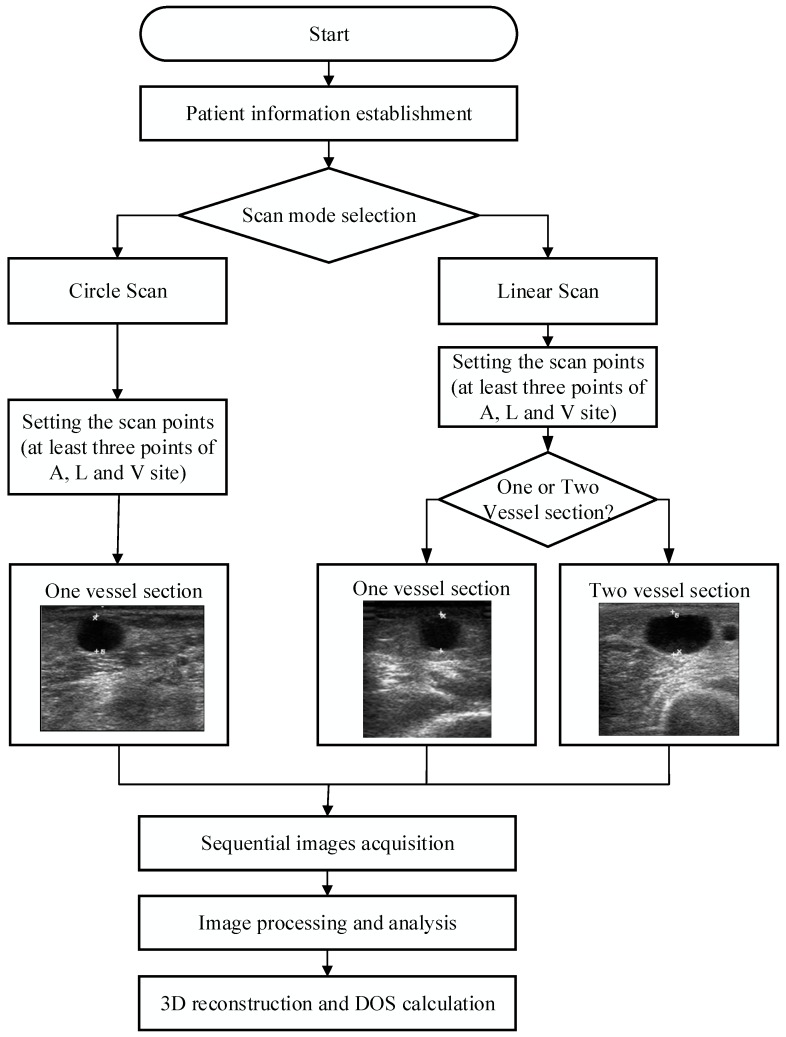
The flowchart of RUS operation and image processing.

After choosing the scan method and related settings, the system calculated the travel path, and then started semi-automatic scanning. When the scan was completed, sequential image processing, which included image smoothing and vessel boundary detection, was executed automatically. Clinicians could adjust the results of the boundary and mark the thickness of the plaque for further processing. Finally, a sequential 2D image could be reconstructed as a 3D model and the DOS calculated for clinical assessment. The system operation flowchart is shown in Figure 5. Because the rectilinear scanning path could not be easily applied by the robotic arm used in this study, after the clinicians set the scan points, the system calculated the path for the arc-like movement. This was done by taking the midpoint of two sets of points in the A, L, and V sites. The system was then able to calculate the path for the arc-like movement after this process. The path coordinate positions were taken and provided to the robotic arm for semi-automatic scanning. The proposed RUS also possessed patient information storage and search functions. We also directly recorded the positions of the occlusion points and stored the related ultrasound images for the convenience of doctors and to facilitate the follow-up of patient conditions.

### 2.7. B-Mode Image Processing and Reconstruction

Ultrasound images had a high noise reflection. Thus, we used a smoothing filter to reduce the noise and gained a clear image [22]. In this study, we used the active contour model (ACM), also known as snakes, for the boundary detection to create the initial contour of vessel section for the reference of clinicians [23,24]. The ACM had the advantage of being capable of encircling the contours of weak or blurred edges efficiently. Secondly, the interior and exterior of the boundaries were circled automatically based on limitation conditions. The ACM was used to locate edges as well as lines. It was capable of retaining the characteristics of contour so as to rebuild contour model of the object completely. The energy function of the ACM was used in this study is shown in Equation (2).

(2) $$E_{snake}=\int_{0}^{1}E_{\mathrm{int}}\left(v\left(s\right)\right)+E_{ext}\left(v\left(s\right)\right)$$

From Equation (2), energy could be categorized as internal energy and external energy, shown as *E*_int_ and *E_ext_*. Contour equation was defined as *v*(*s*) = (*x*(*s*), *y*(*s*)). *s* was a contour of the location parameters and 0 ≤ *s* ≤ 1. *v(s)* was one of the coordinates in the contour. As for the internal energy and external energy with respect to control the shape limitation of ACM, the shape limitation retained the shape characteristic during the process of deformation, and the internal energy function was shown by Equation (3).

(3) $$E_{\mathrm{int}}=\alpha (s){\left|\frac{dv}{ds}\right|}^{2}+\beta (s){\left|\frac{d^{2}v}{ds^{2}}\right|}^{2}$$

In the Equation (3), *α*(*s*) and *β*(*s*) were weighting functions of $\frac{dv}{ds}$ and $\frac{d^{2}v}{ds^{2}}$. *α*(*s*) and *β*(*s*) adjusted influence degree of energy function to further measure the elasticity and stiffness of the snake, respectively. When *α* had a greater value, the contour was better connected during the deformation. However, when *α* had a smaller value, the contour was interrupted. In addition, when *β* had a greater value, the contour became smooth without sharp corners. When *β* had a smaller value, the contour had sharp corners.

The external energy mainly attracted variable models, such as intensity and edge, into the required image characteristic. External energy *E_ext_*(*ν*(*s*)) included energy image *E_image_*(*ν*(*s*)) and energy constraint *E_con_*(*ν*(*s*)). *E_image_*(*ν*(*s*)) was defined as the measure to the attraction of image features such as contours, and *E_con_*(*ν*(*s*)) was defined as the measure of external constrains, which came either from higher-level shape information or other applied energy as shown in Equation (4). In this study, we defined energy constraint was zero.

(4) $$E_{ext}=E_{image}(v(s))+E_{con}(v(s))$$

In addition, external energy image *E_image_*(*ν*(*s*)) could be derived from the image data was shown in Equation (5).

(5) $$E_{image}=\omega_{line}E_{line}+\omega_{edge}E_{edge}+\omega_{term}E_{term}$$

where *ω_line_*, *ω_edge_* and *ω_term_* were appropriate weighting function of the line functional *E_line_*, edge functional *E_edge_* and termination functional *E_term_*, respectively.

**Figure 6:**
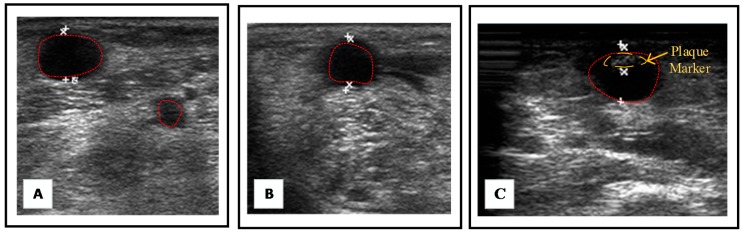
Initial contour of vessel section was created by ACM from (**A**) linear scan; (**B**) circle scan; and (**C**) was an image, which was adjusted contour and marked as plaque by clinicians.

The resulting images with initial contour of vessel section after processing are shown in Figure 6A,B. Afterward, clinicians were able to mark the plaque thickness and in this step, the initial contour could be also justified. The resulting image was shown in Figure 6C. Finally, the proposed system was able to reconstruct a 3D model and calculated the DOS for clinical assessment.

**Figure 7:**
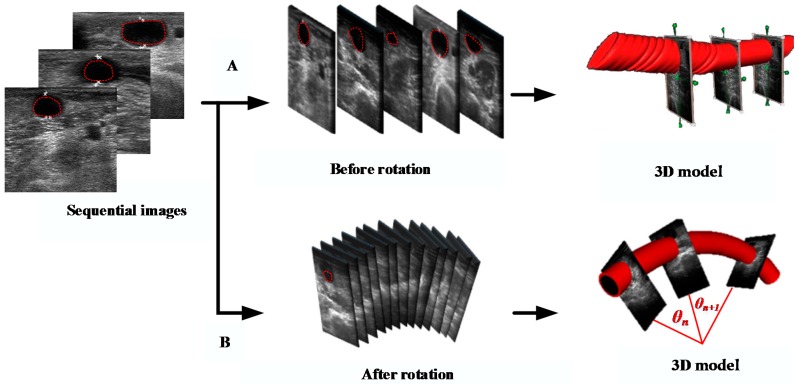
Reconstructed 3D images of the fistula (**A**) before and (**B**) after rotation.

In order to simulate the real fistula position in the space, we had to reconstruct a 3D model using in all the contours in each slice of the image. However, the coordinate system in each image and boundary position had to be rotated firstly along the angle between the ultrasound probe and the center of robotic arm. Arm rotation was made along the *z*-axis; therefore, the *z*-coordinate was not changed at all. The image rotation could be calculated from the *x*- and *y*-coordinates by means of Equations (6). In order to reduce the scan time, the 3D interpolation was used in the study as well as reduced the time of capturing images. The default value was setting as 2 cm. Furthermore, the results of the 3D modeling, before and after image rotation, are shown in Figure 7.

(6) $$\begin{array}{l} x^{\prime }=x\times \cos\theta_{n}+y\times \sin\theta_{n}\\ y^{\prime }=y\times \cos\theta_{n}-x\times \sin\theta_{n}\\ z^{\prime }=z \end{array}$$

where *θ_n_* was the rotation angle around the *z*-axis between two images, and (*x*’, *y*’, *z*’) was the new position of image after performing the rotation in 3D space.

## 3. Experimental Design

### 3.1. Phantom Test

#### 3.1.1. Design of Three Types of Fistula Phantoms

**Figure 8:**
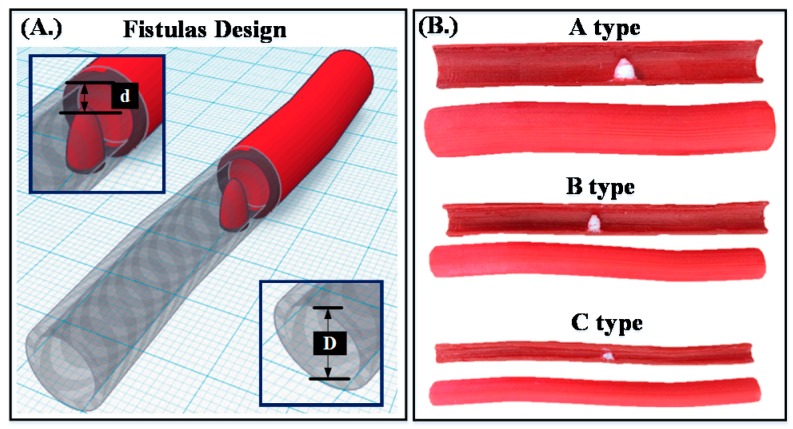
(**A**) Fistula design by Tinker Cad (Autodesk, Inc.). (**B**) Three types of fistula phantom designs.

**Figure 9:**
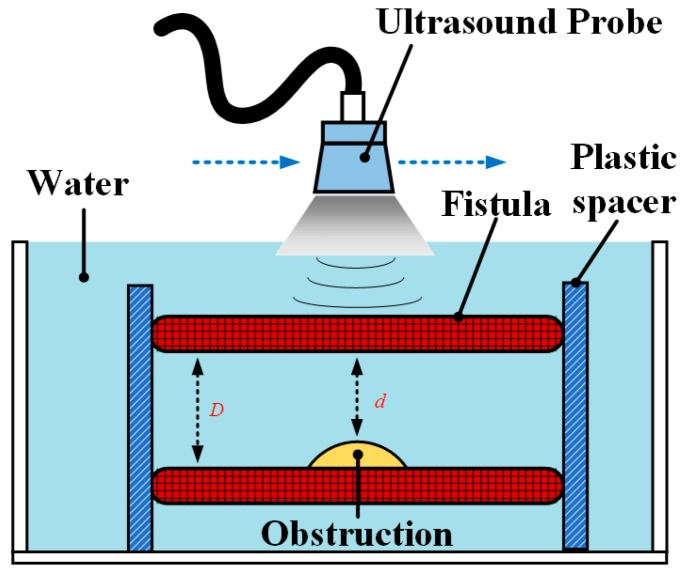
The experimental design of the phantom test by the RUS.

Reviewing previous literature, when the DOS was less than 30%, the fistula had mild stenosis. Under this condition, the life of the fistula could be prolonged through clinical methods, such as avoiding excessive pressure, rehabilitation exercises, or far-infrared light exposure. Thirty percent to 50% DOS was defined as moderate stenosis and could affect, relatively, the efficiency of dialysis. If the DOS was greater than 50%, it was defined as severe stenosis. Once the level of severe stenosis is reached, the patient must undergo fistulization or percutaneous transluminal angioplasty (PTA) to ensure that the blood in the popliteal fistula flows smoothly [25]. Therefore, we designed three types of fistulas with different diameters: A type (*D* = 10 mm); B type (*D* = 8 mm); and C type (*D* = 5 mm) with different levels of stenosis: Class I (*DOS* = 20%); Class II (*DOS* = 40%); and Class III (*DOS* = 80%), as shown in Figure 8. In 2016, Nikitichev, D.I., et al. proposed that by using a light-cured 3D printer with polytetrafluoroethylene to print a blood vessel, a better ultrasound image could be obtained through ultrasound measurements [26]. Therefore, the present study used a light-cured 3D printer (Form1, Formlabs, Somerville, MA, USA) with polytetrafluoroethylene to print the different types of fistulas and scanned the 3D printed fistula using a cone beam computer tomography (CBCT) system to confirm that the printed analog fistulas met our specifications, as shown in Figure 9.

#### 3.1.2. Verification and Comparison of the RUS with the CBCT

**Figure 10:**
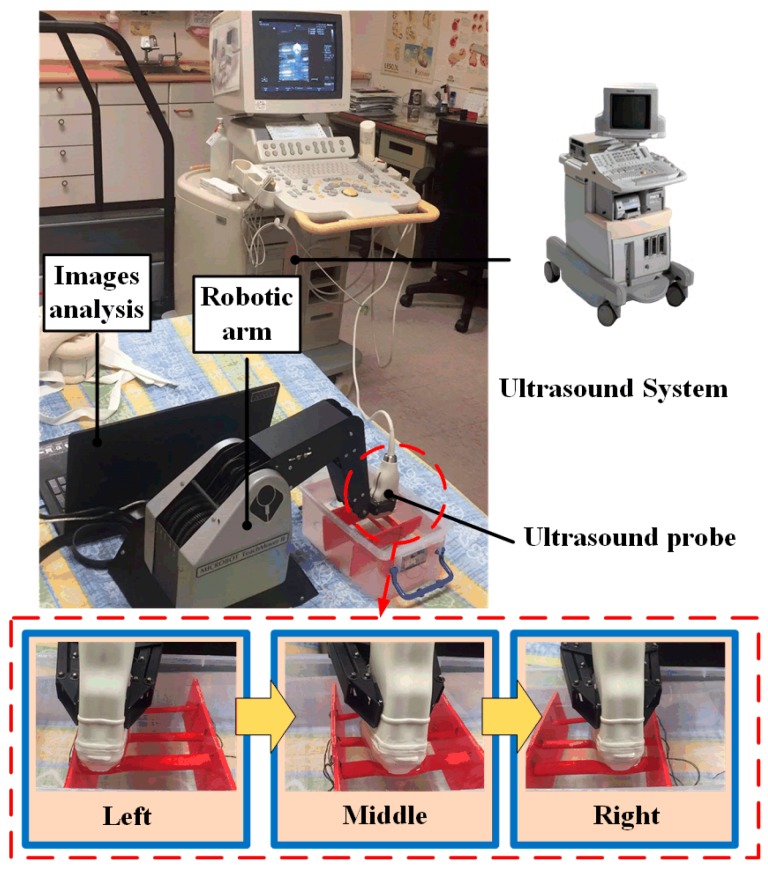
Pictures of the phantom test.

This study placed the phantom, whose position was fixed using a plastic spacer, into a water tank as a simulation of the actual patient fistula condition, shown in Figure 10. The RUS was used to continuously capture sequential ultrasound images for 3D model reconstruction. We also calculated the DOS for system verification and compared them with the results of the DOS measured by the CBCT. The experimental design of the phantom test and corresponding pictures are shown in Figure 10.

### 3.2. Clinical Trials

**Figure 11:**
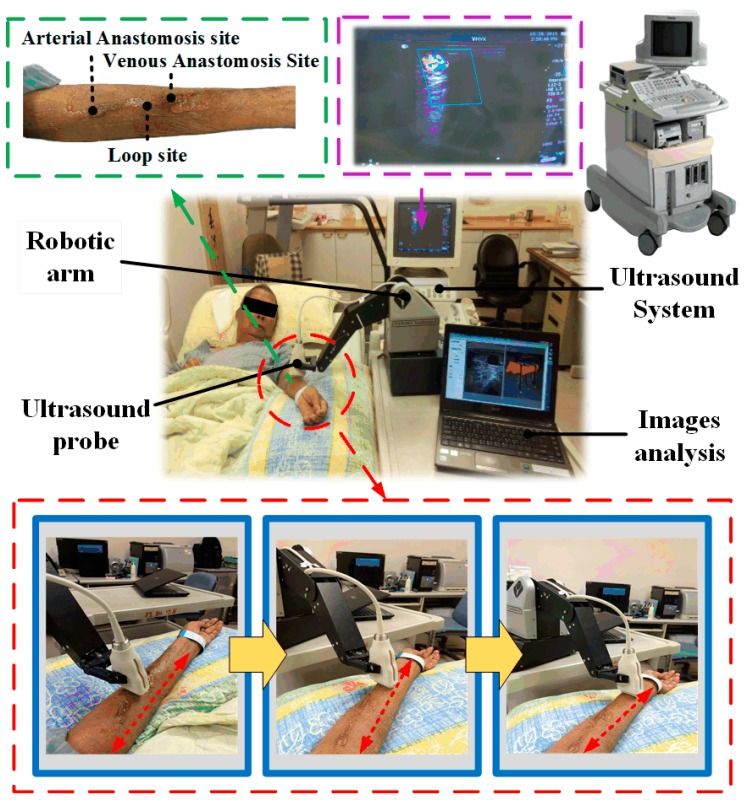
Clinical trials at Kaohsiung Veterans General Hospital (KVGH), Tainan branch, in Taiwan.

In clinical trials, statistical data were obtained by screening patients with the following characteristics: (a) autologous AVF at 8 weeks post creation or AVG at 6 weeks post creation; (b) not restless; and (c) with no surface injury on the arm used during dialysis or infection risk. Ten patients were recruited in this study with a mean age of 69.4 ± 13.1 years. The clinical trial pictures taken from Kaohsiung Veterans General Hospital (KVGH), Tainan branch, under the institutional review board (IRB): 17-CT3-11(170202-1) are shown in Figure 11. Before the trials, clinicians measured the DOS through a traditional ultrasound imaging system. Both the DOS results and the operation time were recorded for comparison with the proposed RUS results in the next step. Afterwards, the clinician, who is one of the coauthors of this paper, tried to find the best setting for the RUS by trying out various starting positions, scan methods, and speed of arm in order to acquire stable images at a steady rate. In this study, the clinicians performed circle and linear scans on four and six patients, respectively. Each scan was repeated ten times for each patient, and the average of the related parameters was obtained to reduce the variation and white noise effect.

## 4. Results

### 4.1. Results of Phantom Test

In order to confirm whether the level of stenosis of the proposed phantom was the same as the design specification, the study used CBCT to carry out some measurements. The results are shown in Table 1. It could be observed that the average error in types A and B was only between 1.3% and 2.4%. However, the error in the smaller diameter, type C, was relatively high, up to 4.4%. All of the phantoms did not exceed the original specification of the DOS range confirmed by the CBCT. In addition, the comparison between the CBCT and the RUS scan showed that the calculated stenosis values were all less than 3%. Similarly, the error in the small diameter, type C, was relatively large, up to 2.7%. The experimental results show that the proposed RUS system could successfully analyze the DOS and effectively classify the DOS into three levels in the phantom test within the set of vascular size and the range of stenosis.

### 4.2. Results of Clinical Trials

**Figure 12:**
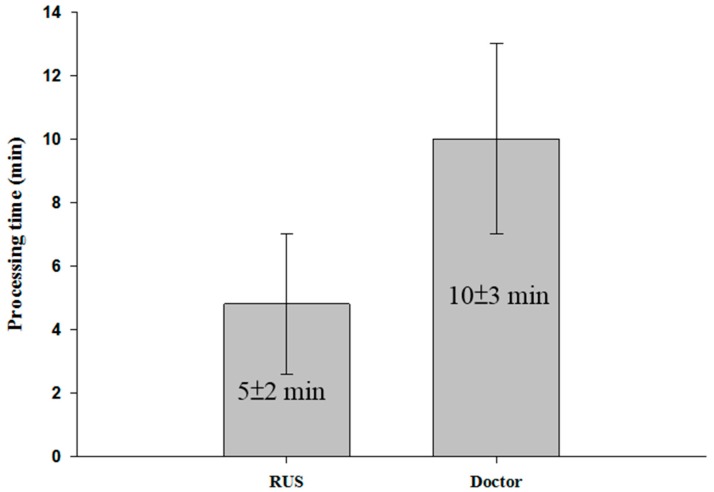
Comparison of processing time.

Two consecutive scans of ten patients were made. The error in the measurement made by clinicians and the RUS was less than 10%. Moreover, the parameter of R-square in the linear regression was greater than 0.95. Detailed data for the tests are shown in Table 2. We also found that average error of results in several patients was larger than the error measured in others. This might be because the scan path followed by the RUS did not cover all the spots in the fistula scanned using the traditional way of scanning. During clinical examinations, clinicians usually focus on the A or V sites of the fistula to find the larger DOS point. However, in some cases, the major DOS of patients who have a multi-point stenosis may not occur around sites A or V [27]. When a complicated stenosis situation was encountered, the measurement made by clinicians produced a larger error. This might be the major reason for the large difference when comparing measurement results made by clinicians and the RUS. Based on the above process, it took an average of 13 min for each physical examination. However, the proposed RUS just took an average of 7 min. The time comparison is shown in Figure 12.

## 5. Conclusions and Discussion

In the phantom experiment, the light-curing 3D printer had high forming accuracy because each layer of resin was solidified and covered with a new layer by moving the workbench to smooth and stabilize the deformation of the phantom. Therefore, the error of the DOS in the phantom was less than 4.1% when compared with what was confirmed by the CBCT scan. However, the type C fistula showed a greater error when compared with the others. It was observed that the smaller blood vessel phantom was easily affected by vibration during the printing process. Since the diameter of the type C fistula was only 5 mm, smaller than the others, it had a relatively higher DOS error. In this study, it was confirmed that the designed phantom had a good clinical reference value and could be applied to related studies.

In the actual measurement of ten patients during clinical procedures, the present RUS could effectively save 46% of the time during examinations. The measurement error between the RUS and clinicians was less than 10% and had a higher correlation. In some cases, the average error was larger than in others. This may be because the RUS scan path did not cover all the fistula spots covered by traditional ultrasound. For patients who have a multi-point stenosis, the major DOS does not usually occur around at the A or V sites [27]. The study also found that the average variation in the ten repeated measurements obtained from clinicians and the RUS was 8.4% and 1.6%, respectively. The high and reproducible error occurred due to the difference in the position of the traditional way of scanning and the RUS. In addition, the stability of the acquired ultrasound image was affected by slight shaking of the hand. The results indicated that the proposed RUS had a high reproducibility and accuracy. This could provide more objective results and increase work efficiency during clinical assessment.

Moreover, during clinical trials, when a patient had a fistula aneurysm, the RUS measurement became difficult because an aneurysm could cause an uneven scanning path on the skin of the hand. In this situation, the robotic arm had difficulties fixing the ultrasound probe completely on the uneven skin surface. In the future, computer vision and pressure sensing function could be combined with the proposed RUS to check the pressure and the *z*-axis of the probe. If the probe is not properly fixed on the skin, the *z*-axis of the robotic arm can be adjusted, and the pressure can be monitored to prevent overexertion of pressure on the fistula. The scan images, 3D model, and related parameters of individual patients from the proposed RUS can be provided for clinical assessment. They can also be used as a reference to track the status of the fistula. The proposed system has a high potential to be used as an auxiliary tool in relevant clinical applications.

## Figures and Tables

**Table 1:** Comparison table for phantom test. CBCT = cone beam computer tomography.

Phantom Test												
		Design			CBCT			RUS			Error (%)	
***Type/Class***		***d***	***D***	***DOS***	***d***	***D***	***DOS***	***d***	***D***	***DOS***	**Design and CBCT**	**CBCT and RUS**
A	I	9	10	19.0%	8.8	9.8	20.6%	8.7	9.8	21.2%	1.6%	0.6%
	II	7.8	10	40.2%	7.5	9.8	42.6%	7.3	9.7	43.4%	2.4%	0.7%
	III	4.5	10	80.2%	4.3	10	81.5%	4.2	9.8	81.6%	1.3%	1.0%
B	I	7	8	23.0%	7	7.9	21.5%	7.1	8.1	23.2%	1.5%	1.7%
	II	6.2	8	40.0%	6.2	7.9	38.4%	6	7.8	40.8%	1.6%	2.4%
	III	3.5	8	80.9%	3.3	7.8	82.2%	3.1	7.9	84.6%	1.3%	2.4%
C	I	4.5	5	19.0%	4.2	4.8	23.4%	3.9	4.5	24.9%	4.4%	1.5%
	II	3.9	5	39.2%	3.6	4.8	43.3%	3.5	4.7	44.5%	4.1%	2.2%
	III	2.2	5	80.6%	1.8	4.6	84.7%	1.6	4.5	87.4%	4.1%	2.7%

*D* unit is mm, *d* unit is mm, *DOS* means by Equation (1).

**Table 2:** Comparison of DOS measurement by RUS and doctors.

	Measured by RUS				Measured by Doctor				Average Error (%)
Patient	*D*	*d*	*DOS_RUS_*	Variation	*D*	*d*	*DOS_Doc_*	Variation	
1	76.8	23.6	90.6	1.2%	75.2	19.8	93.0	4.7%	2.4
2	88.3	44.4	74.7	0.6%	86.8	41.7	76.9	6.8%	2.9
3	128.0	96.0	43.8	1.8%	125.7	88	50.9	17.1%	7.1
4	108.0	85.1	37.9	2.1%	107.2	81.8	41.8	9.6%	3.9
5	96.1	81.2	28.6	1.1%	94.8	78.4	31.6	8.8%	3.0
6	170.0	143.4	28.8	2.2%	169.8	140.3	31.7	12.9%	2.9
7	93.2	77.2	31.3	1.4%	90.1	74.2	32.1	7.0%	2.5
8	113.0	92.8	32.6	1.6%	109.2	85.1	39.2	11.1%	6.6
9	98.5	79.8	34.4	1.5%	97.8	73.4	43.7	3.6%	9.3
10	96.0	52.2	70.4	2.0%	93.1	48.6	72.7	2.4%	2.3

*D* and *d* unit is mm and *DOS_RUS_* and *DOS_Doc_* unit is %. *D*, *d*, *DOS_RUS_*, and *DOS_Doc_* refer to the average number that was calculated in ten measurements. Variation was calculated by measuring the *DOS_RUS_* and *DOS_Doc_* ten times. Average error was calculated by *DOS_RUS_*/*DOS_Doc_*.
